# Comparing planning time, delivery time and plan quality for IMRT, RapidArc and tomotherapy

**DOI:** 10.1120/jacmp.v10i4.3068

**Published:** 2009-10-07

**Authors:** Mike Oliver, Will Ansbacher, Wayne A. Beckham

**Affiliations:** ^1^ Department of Medical Physics British Columbia Cancer Agency Victoria British Columbia Canada

**Keywords:** IMRT, RapidArc, tomotherapy, treatment planning

## Abstract

The purpose of this study is to examine plan quality, treatment planning time, and estimated treatment delivery time for 5‐ and 9‐field sliding window IMRT, single and dual arc RapidArc, and tomotherapy. For four phantoms, 5‐ and 9‐field IMRT, single and dual arc RapidArc and tomotherapy plans were created. Plans were evaluated based on the ability to meet dose‐volume constraints, dose homogeneity index, radiation conformity index, planning time, estimated delivery time, integral dose, and volume receiving more than 2 and 5 Gy. For all of the phantoms, tomotherapy was able to meet the most optimization criteria during planning (50% for P1, 67% for P2, 0% for P3, and 50% for P4). RapidArc met less of the optimization criteria (25% for P1, 17% for P2, 0% for P3, and 0% for P4), while IMRT was never able to meet any of the constraints. In addition, tomotherapy plans were able to produce the most homogeneous dose. Tomotherapy plans had longer planning time, longer estimated treatment times, lower conformity index, and higher integral dose. Tomotherapy plans can produce plans of higher quality and have the capability to conform dose distributions better than IMRT or RapidArc in the axial plane, but exhibit increased dose superior and inferior to the target volume. RapidArc, however, is capable of producing better plans than IMRT for the test cases examined in this study.

PACS number: 87.55.x, 87.55.D, 87.55.de, 87.55.dk

## I. INTRODUCTION

Intensity‐modulated radiation therapy (IMRT), intensity‐modulated arc therapy (IMAT) and tomotherapy (Tomo) are all advanced external beam radiation therapy treatment techniques that have been implemented for routine clinical use at different time points over the last 10 years.^(1)^ IMRT using a conventional linear accelerator equipped with a multi‐leaf collimator (MLC) was adapted for clinical use to treat prostate cancer in 1995 (despite the fact that IMRT using compensators was performed earlier).^(2)^ This was followed by the implementation of the technique for other treatment anatomical locations.^(^ ^3^ ^,^ ^4^ ^)^ A recent meta‐analysis examined data from 56 clinical trials has suggested that IMRT can reduce toxicities as compared to non‐IMRT treatments; however, the data regarding local control and overall survival are inconclusive.^(5)^ With emerging data demonstrating the advantages of IMRT, future advancements in advanced radiation therapy delivery will include improvements in quality, efficiency, accuracy with image guidance, and ability to paint dose distributions.^(1)^ This paper investigates both improvements in quality and delivery efficiency of radiation therapy.

IMAT implemented with the gantry of the linear accelerator rotating during delivery along with MLC variations, was first proposed by Yu.^(6)^ Clinical implementation of arc therapy techniques was sparse, but treatments were performed for central nervous system, prostate, head and neck, whole abdominopelvic treatments, rectal cancer and endometrial cancers.^(^ ^7^ ^–^ ^9^ ^)^ A major advance in IMAT was realized when Otto implemented his volumetric modulated arc therapy (VMAT) algorithm.^(10)^ VMAT uses a progressive sampling algorithm which starts with coarse gantry samples and then, throughout the optimization, the arc resolution is gradually improved. Without this algorithm, neighboring segments are highly restricted by the allowed leaf motion. VMAT obviates this restriction by allowing large leaf movements early in the optimization and more restricted leaf motion in the later stages.^(10)^ The optimization time is also greatly reduced. Otto's algorithm has been implemented by Varian (Varian Medical Systems, Palo Alto, CA, USA) and is marketed as RapidArc. In this implementation, the progressive sampling is achieved through five discrete “multi‐resolution” (MR) levels in which the number of segments increases from 10 to 177. Elekta (Elekta AB, Stockholm, Sweden) also have a product named VMAT which does not implement Otto's algorithm but uses a proprietary algorithm.^(11)^ Both Varian's and Elekta's implementations of arc therapy allow for dose rate variations.

Helical tomotherapy was first proposed by Mackie and is now commercially available from TomoTherapy (TomoTherapy Inc, Madison, WI, USA).^(12)^ Tomotherapy is a technique whereby a fan beam of radiation rotates around the patient who is translated through the bore of the tomotherapy machine as in conventional computed tomography. The beam trajectory follows a helical path during delivery and is modulated by a binary MLC. Treatments are optimized from 51 projections and can be conceptualized as IMRT beams delivered from 51 equally spaced angles.^(13)^ The first patient treated with helical tomotherapy was in 2002 and, since then, units have been sold worldwide.^(14)^

There is a need to characterize the differences in plan quality, planning time, and delivery time for IMRT, modern arc therapy, and tomotherapy. Previous studies have compared IMRT with tomotherapy, IMRT with arc therapy, and tomotherapy with arc therapy.^(^ ^8^ ^,^ ^15^ ^–^ ^21^ ^)^ There is a single study that examines IMRT, arc therapy, and tomotherapy planning for five patients with benign intracranial lesions.^(22)^ The conclusions of this study are that all techniques are practically equivalent. The study recommends providing more challenging cases to evaluate the treatment techniques, and provides the primary motivation for this study. Furthermore, a recent publication by Bortfeld and Webb provided some theoretical considerations when considering the quality of dose distributions that can be achieved for IMRT, single arc IMRT, and tomotherapy based on a 2D phantom with an analytically derived solution.^(23)^ They conclude that a single arc that is delivered in less than 2 minutes may unduly compromise the plan quality for very complex cases, and feel that the plan quality for IMRT and single arc‐IMRT should be similar.

The goal of this study is to create IMRT, RapidArc and tomotherapy treatment plans for four virtual phantoms including the Brahme phantom^(23)^ and compare the treatment plan quality, optimization time and delivery time.

## II. MATERIALS AND METHODS

### A. overview of methods

**Figure 1:**
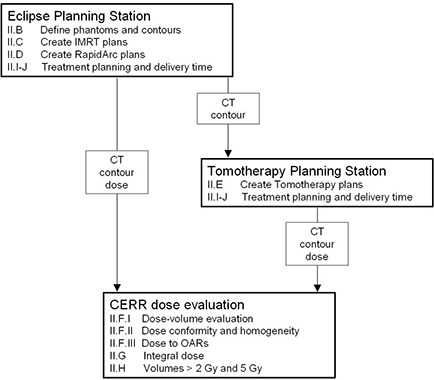
A block diagram of the different major pieces of software used in this study, along with the sections of the methods where these points are further explained and the data that is transferred, listed either as an incoming or outgoing arrow from that module.

A summary of the methodology for this paper has been included in Fig. 1 which refers to the different section headings, the major planning systems, and dose evaluation software that is used in this study. Data transferred between systems is listed either as an input or output arrow in Fig. 1.

### B. Definitions of phantoms, contours and plan objectives

**Figure 2:**
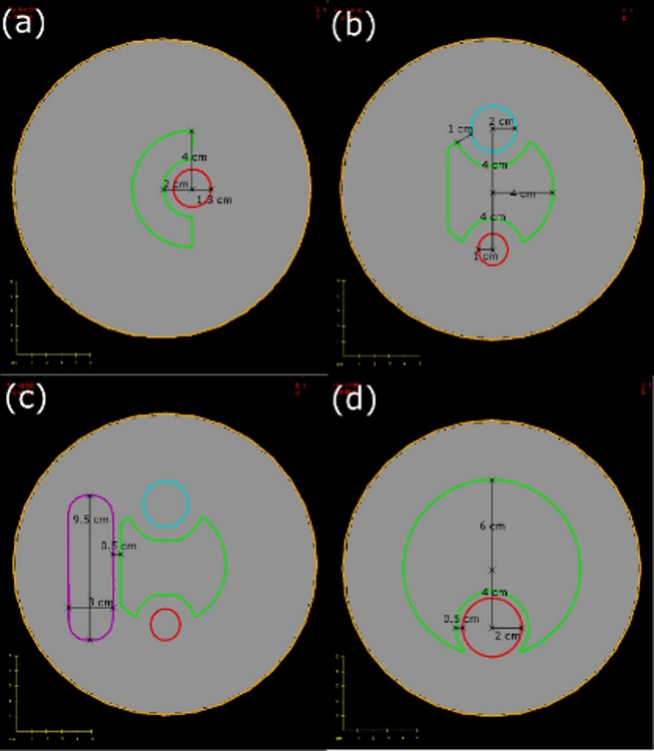
Axial slices of all phantoms with PTV and OARs shown: (a) Phantom1 with PTV (green) and OAR1 (red); (b) Phantom 2 with PTV (green), OAR1 (red) and OAR2 (blue); (c) Phantom 3 with PTV (green), OAR1 (red), OAR2 (blue) and OAR3 (purple); (d) Phantom 4 with PTV (green) and OAR (red).

All virtual phantoms and contour sets were created within the Eclipse treatment planning system (Eclipse 8.6 pre‐release version with RapidArc planning capability, Varian Medical Systems, Palo Alto, CA). All phantoms were water equivalent and had a 10 cm radius and a length of 25.25 cm. An axial view of all of the phantoms including contours is provided in Fig. 2. Each phantom included an external contour, a planning target volume (PTV), and a number of organs at risk (OARs). The contours were extended in the third dimension by varying amounts. The superior‐inferior lengths of the PTVs and OARs are as follows: PTV length is 8.5 cm, OAR1 length is 6 cm for Phantom 1; PTV, OAR1, OAR2, OAR3 length is 5.25 cm for Phantoms 2 and 3; PTV length is 8 cm, OAR length is 7.5 cm for Phantom 4. The locations of the PTV and OARs were chosen because they represent geometries that are seen in the clinic or are well‐established test cases. Phantom 1 is similar to the American College of Radiology test case.^(24)^ Phantom 2 represents a prostate geometry, and Phantom 3 represents a head and neck geometry. Finally, Phantom 4 was the test case that was used in the analysis by Bortfeld.^(23)^

Planning objectives for all phantoms are listed in Table 1. The prescription dose for these treatment plans was 60 Gy in 30 fractions. The dose‐volume objectives were chosen to represent a significant challenge to each of the three techniques. The choice of importance values varied depending on the planning system that was used.

**Table 1:** A listing of all dose volume criteria used during optimization of Phantoms 1–4.

	*PTV*		*OAR1*			*OAR2*			*OAR3*		
	*Min Dose (Gy)*	*Max Dose (Gy)*	*DVH Vol (%)*	*DVH Dose (Gy)*	*Max Dose (Gy)*	*DVH Vol (%)*	*DVH Dose (Gy)*	*Max Dose (Gy)*	*DVH Vol (%)*	*DVH Dose (Gy)*	*Max Dose (Gy)*
Phantom 1	60.0	65	50.0	13.3	20.0						
Phantom 2	60.0	65.0	50.0	10.0	20.0	50.0	10.0	20.0			
Phantom 3	60.0	65.0	50.0	10.0	20.0	50.0	10.0	20.0	50.0	15.0	30.0
Phantom 4	60.0	65.0	50.0	15.0	30.0						

### C. Sliding window IMRT planning

Sliding window IMRT treatment plans were created within the Eclipse planning environment. Two plans were created consisting of either 5 (IMRT5) or 9 (IMRT9) beams using the 120 leaf Millennium MLC which has a 5 mm leaf width at isocenter. The choice of 5 or 9 beams represents a trade‐off between treatment time and plan quality. It has been shown that for more than 7–9 beams, the choice of beam directions does not matter for coplanar IMRT deliveries.^(^ ^25^ ^,^ ^26^ ^)^

Optimization was performed in “beamlet” mode with approximately 250 iterations performed before the final dose was calculated. After 250 iterations, the cost function has converged for all plans. The final dose matrix was calculated with the Analytical Anisotropic Algorithm (AAA, version 8.6.10) using a voxel size of $0.25\times 0.25\times 0.25\;{\text{cm}}^{3}$. The CPU within Eclipse is a dual Intel Xeon quad‐core processor running at 2.50 GHz.

### D. RapidArc planning

RapidArc plans were created in the same Eclipse system, which supports planning with more than one arc. Single 360° rotation RapidArc (RA1) and dual 360° rotation RapidArc (RA2) treatment plans were created with the collimator rotated to 45°. The implementation of RapidArc with either 1 or 2 arcs represents a trade‐off between plan quality and treatment time whereby single arc plans are expected to be deliverable in a short period of time. The consequence of adding an additional arc may improve plan quality with an increase in the treatment time. A technique was developed to initialize the MLC positions of all arc segments to the PTV outline minus the OARs. In this version of the RapidArc planning system, the optimization appears to switch to the next MR level before the objective function has converged for a given level. The switch can be delayed by the user, and this was done progressively for 1 minute at MR1 up to 5 minutes at MR5. Upon completion of the “Arc Optimization”, the progress of the optimization was saved and the optimization was then “Continued” at resolution level five for 5 additional minutes. The final dose calculation was computed with AAA and a voxel size of $0.25\times 0.25\times 0.25\;{\text{cm}}^{3}$. The hardware specifications for the Eclipse system are the same as described in the previous section.

### E. Tomotherapy planning

Tomotherapy treatment plans were created using the TomoTherapy planning station version 3.1.2.3. In tomotherapy planning, the field width is defined as the axial thickness of the fan beam, the pitch is defined as the couch travel distance for a complete gantry rotation relative to the axial beam width at the axis of rotation, and the modulation factor is defined as the maximum leaf opening time divided by the average leaf opening time. The pitch factors were chosen to reduce the thread effect on the final dose distribution and were chosen to be 0.86/n, where *n* is an integer.^(27)^

Treatment planning consisted of performing a full beamlet dose calculation following 250 optimization iterations. After 250 iterations the cost function has converged for all plans. The tomotherapy plans (TOMO) were created with a fan beam width of 2.5 cm at isocenter, a pitch of 0.287, and a modulation factor of 2.5. The dimensions of the dose voxels of tomotherapy treatment plans were $0.31\times 0.31\times 0.25\;{\text{cm}}^{3}$. The specifications for the tomotherapy clusters are 16 Intel dual processors running at 2.4 GHz.

### F. Plan quality

In this study, the primary metric of plan quality was the ability to meet dose volume criteria during optimization as a binary metric as described below in F.1. The ability to meet DVH objectives can be used along with dose conformity and homogeneity and mean dose to the OARs as described in sections F.2 and F.3 below, respectively.

#### F.1 Dose volume evaluation

Upon completion of optimization, all plans were brought into the Computational Environment for Radiotherapy Research (CERR version 3.1) for dosimetric evaluation.^(28)^ All plans were optimized such that 95% of the volume of the PTV received a dose of 61 Gy.

**Figure 3:**
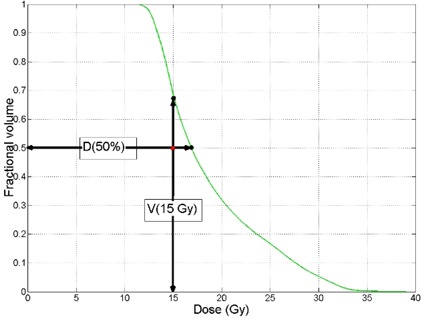
The red dot on the plot represents the desired dose‐volume objective and the green curve demonstrates the optimized dose volume histogram (DVH) for the organ. In this case the desired dose is 15 Gy to 50% of the volume. The values that are reported in Table 4 are the dose to 50% volume and volume receiving 15 Gy.

The minimum dose is reported as the dose to 99.5% of the PTV and the maximum dose is reported as the dose to 0.5% of the PTV. Dose‐volume criteria, listed in Table 1, are reported as the percent volume receiving dose D, $\left(V_{D}\right)$ and dose to a minimum percent volume V $\left(D_{v}\right)$. Figure 3 is a graphical representation of how the dose‐volume constraints are reported. A binary metric which includes the ability to meet DVH criteria was used for evaluation of treatment plans.

#### F.2 Conformity and homogeneity indices

The radiation conformity index (RCI) and dose homogeneity index (DHI) of the resultant treatment plans were computed using Eqs. 1 and 2, respectively. The RCI describes the volume within the PTV that receives the prescription dose or higher $\left(V_{D,\text{PTV}}\right)$ divided by the volume within the external body contour that receives the prescription dose or higher $\left(V_{D,\text{Body}}\right)$ and provides a metric to determine how close the prescription isodose volume is spread within the PTV and normal tissue.

(1) $$\text{RCI}=\frac{V_{D_{P,},\text{PTV}}}{V_{D_{P,},\text{Bod}y}}$$

The DHI describes the uniformity of the dose within the planning target volume and is a ratio of the minimum dose ${(D}_{D.99\%})$ to the maximum dose ${(D}_{D.0\%})$.

(2) $$\text{DHI}=\frac{D_{99.5\%}}{D_{0.5\%}}$$

#### F.3 Mean dose to the OARs

The mean dose to each of the OARs was also recorded. The volume points that were desired were the dose to 50% of the volume and the maximum dose to the OAR; therefore, the mean dose should be correlated with the desired dose‐volume points.

### G. Integral dose

The integral dose is reported as the sum of all dose voxels times their mass, as shown in Eq. 3. The phantom has unit density, so the equation can be simplified as shown below, where *N* is the number of voxels, $D_{\text{mean}}$ the mean dose to the body contour, and $m_{\text{voxel}}$ the mass of a voxel. The simplest form of the equation is the mass of the body of the phantom $\left(m_{\text{body}}\right)$ times the mean dose to the body of the phantom ${(D}_{\text{mean},\text{body}})$.

(3) $$E_{\text{Int}\text{egr}al}=\sum_{i=1}^{N}D_{i}\cdot m_{i}=N\cdot D_{\text{mea}n}\cdot m_{\text{vox}el}=m_{\text{bod}y}\cdot D_{\text{mea}n,\text{bod}y}$$

### H. Volume receiving >2Gy and >5Gy

Some models of radiation carcinogenesis suggest that the dose‐response relationship is linear up until a dose of 6 Gy, where it then reaches a plateau.^(^ ^29^ ^,^ ^30^ ^)^ The volumes receiving greater than 2 Gy and greater than 5 Gy would be important in this context and are reported for each treatment plan.

### I. Estimation of treatment planning times

The treatment planning time is defined as the time from starting a plan until final optimization and dose calculation are completed. It is assumed that the plan can be delivered clinically and therefore would include selecting the relevant parameters, optimizing the delivery, and performing a final dose calculation.

### J. Estimation of treatment delivery time

The estimated treatment delivery time will be defined as the time from first beam on until the last beam is turned off. For sliding window IMRT, it is the dose rate multiplied by the number of MU per field, plus the time that the gantry takes to rotate between successive fields, plus a parameter “delta” which takes into account the time for mode up, data transfer of the MLC delivery files, error in the estimated rotation time, and operator reaction time. The delta parameter was not added for the first beam because the data transfer and mode up happens before the first beam. Delta was determined (from 215 head and neck IMRT fields delivered at our center) as the difference between the theoretical beam‐on time at 400 MU/min plus gantry rotation at 360°/min and the actual delivery time as recorded by the record and verify system. For RapidArc, the estimated treatment delivery time is the sum of the time spent at each of the 177 segments within an arc. The time is the sum of all angular increment per segment divided by the gantry rotation rate. For multiple arc plans, the delta parameter was added for the second arc because all data transfer happens before the beam is turned on. The estimated treatment delivery times for tomotherapy are calculated by the planning system and recorded from the final plan report.

## III. RESULTS

### A. dose‐volume evaluation

**Figure 4:**
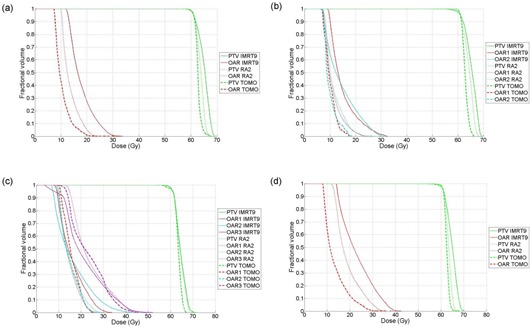
Dose volume histograms for 9‐field IMRT (IMRT9, solid), 2‐arc RapidArc (RA2, dotted) and tomotherapy (TOMO, dash dotted) plans delivered to Phantom 1 (a), Phantom 2 (b), Phantom 3 (c) and Phantom 4 (d).

**Figure 5:**
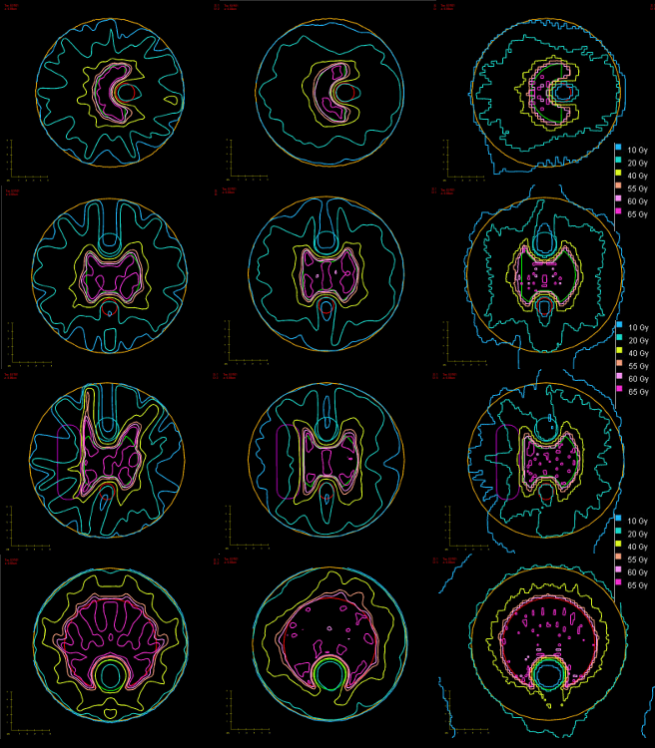
Axial dose distribution of Phantoms 1 (first row), 2 (second row), 3 (third row), and 4 (fourth row) for 9‐field IMRT (left), dual arc RapidArc (center), and tomotherapy (right) with isodose lines at 65, 60, 55, 40, 20 and 10 Gy.

The dose‐volume evaluation was performed, and data from all treatment plans is presented in Table 2. Representative DVH curves for the 9‐field IMRT, 2‐arc RapidArc, and tomotherapy plans are shown in Fig. 4. Figure 5 shows isodose lines taken for axial slices from the 9‐field IMRT, dual arc RapidArc, and tomotherapy dose distributions. Based on the data from Table 2, Phantom 1 had 0/4 optimization constraints met for IMRT5 and IMRT9, 1/4 constraints met for RA1 and RA2 and 2/4 for TOMO; Phantom 2 had 0/6 optimization constraints met for IMRT5, IMRT9 and RA1, 1/6 constraints met for RA2, and 4/6 for TOMO. For Phantom 3, all of the plans had 0/8 constraints met. Finally, for Phantom 4, only TOMO had 2/4 constraints met and the rest of the plans had 0/4 constraints met.

**Table 2:** A summary of all of the dose and volume parameters along with the actual dose or volumes that have been attained are listed. As a reference, the desired dose and volume values are listed in bolded text above the attained values. Values that are bolded and italicized are parameters that met the predefined optimization criteria.

		*PTV*		*OAR1*			*OAR2*			*OAR3*		
		*Min Dose*	*Max Dose*	*DVH ‐ Vol*	*DVH Dose*	*Max Dose*	*DVH ‐ Vol*	*DVH ‐ Dose*	*Max Dose*	*DVH ‐ Vol*	*DVH ‐ Dose*	*Max Dose*
Phantom 1		60	65	50	13.3	20						
IMRT	5 field	59.2	68.9	82.1	16.0	29.3						
	9 field	59.3	73.1	87.1	16.4	30.3						
RapidArc	1 arc	59.2	68.7	40.9	12.4	23.6						
	2 arc	59.5	67.0	40.1	12.5	22.6						
Tomo		59.1	67.2	15.1	9.4	17.0						
Phantom 2		60.0	65.0	50.0	10.0	20.0	50.0	10.0	20.0			
IMRT	5 field	58.2	69.3	97.7	13.2	28.6	63.8	25.3	27.6			
	9 field	57.0	69.2	90.4	13.1	31.6	64.8	25.7	29.5			
RapidArc	1 arc	58.1	68.7	58.5	10.3	23.4	52.3	10.2	24.1			
	2 arc	58.3	68.0	50.2	10.0	23.1	47.3	9.8	22.9			
Tomo		59.9	66.1	42.6	9.5	17.4	33.9	8.8	17.9			
Phantom 3		60.0	65.0	50.0	10.0	20.0	50.0	10.0	20.0	50.0	15.0	30.0
IMRT	5 field	58.8	70.4	79.6	12.9	28.4	66.1	13.3	35.4	66.9	19.5	44.7
	9 field	58.3	70.1	84.8	14.3	31.3	69.4	13.6	40.1	69.7	18.7	46.8
RapidArc	1 arc	58.3	69.0	94.9	13.4	24.7	81.9	13.1	28.6	91.3	21.7	44.2
	2 arc	58.3	69.0	90.7	13.5	26.1	88.1	13.2	27.8	93.8	21.2	43.6
Tomo		56.7	66.7	99.9	15.1	24.6	96.8	13.5	25.2	83.0	21.5	43.6
Phantom 4		60.0	65.0	50.0	15.0	30.0						
IMRT	5 field	55.0	75.3	86.5	23.3	41.4						
	9 field	57.6	69.5	87.6	21.6	39.4						
	2 arc	59.0	66.5	68.3	16.9	33.8						
Tomo		57.4	65.6	28.7	11.2	29.5						

### B. Radiation conformity index, dose homogeneity index

A complete list of the radiation conformity and dose homogeneity indices for all plans is included in Table 3. The average RCI values were 0.87±0.07 for IMRT, 0.91±0.02 for RapidArc, and 0.81±0.07 for tomotherapy. The average DHI values were 0.83±0.03 for IMRT, 0.86±0.02 for RapidArc, and 0.88±0.02 for tomotherapy.

**Table 3:** Radiation conformity index and dose homogeneity index for all IMRT, RapidArc and tomotherapy plans delivered to the test phantoms are reported in the first two columns. The mean dose to the OARs (units of Gy) is listed in the third, fourth and fifth columns. The integral dose (units of Joules), volume receiving greater than 2 Gy, and volume receiving greater than 5 Gy, (in cc) are reported in the sixth, seventh and eighth columns, respectively.

			*RCI*	*DHI*	*Mean OAR1*	*Mean OAR2*	*Mean OAR3*	*Integral Dose*	V>2Gy	V>5Gy
Phantom	IMRT	5 field	0.855	0.860	17.32			89.0	4148	3275
1		9 field	0.913	0.811	17.78			87.8	4151	3425
	RapidArc	1 arc	0.890	0.862	13.65			85.8	4338	3379
		2 arc	0.914	0.888	13.53			85.7	4311	3381
	Tomo		0.706	0.880	10.47			106.9	4884	4281
Phantom	IMRT	5 field	0.876	0.839	14.66	13.88		59.2	2617	2051
2		9 field	0.921	0.824	14.90	14.28		59.0	2662	2101
	RapidArc	1 arc	0.911	0.846	11.63	11.68		58.0	3019	2181
		2 arc	0.926	0.857	11.20	11.19		57.2	2970	2158
	Tomo		0.793	0.906	10.07	9.93		75.2	3507	2995
Phantom	IMRT	5 field	0.758	0.835	14.55	15.16	21.26	59.1	2630	2035
3		9 field	0.783	0.832	15.86	16.17	21.46	58.3	2633	2031
	RapidArc	1 arc	0.904	0.844	14.44	14.56	24.27	58.6	3104	2226
		2 arc	0.911	0.846	14.43	14.71	23.81	58.2	3060	2223
	Tomo		0.847	0.850	15.74	14.77	23.23	73.5	3510	2977
Phantom	IMRT	5 field	0.769	0.730	24.34			148.3	4991	3477
4		9 field	0.912	0.829	23.06			142.3	4833	3450
	RapidArc	1 arc	0.904	0.826	20.24			142.1	5562	3915
		2 arc	0.940	0.887	18.81			141.2	5629	3915
	Tomo		0.900	0.874	13.26			158.9	5367	4195

### C. Mean dose to the OARs

The mean dose values for each phantom and delivery technique are listed in Table 3. The general trend regarding the mean dose to the OARs for Phantoms 1, 2 and 4 is $d_{\text{OAR},\text{TOMO}}{<d}_{\text{OAR},\text{RA}2}{<d}_{\text{OAR},\text{RA}1}{<d}_{\text{OAR},\text{IMRT}5\text{or}9}$.

### D. Integral dose and volumes receiving in excess of 2 and 5 Gy

The integral doses, in Joules, are listed in Table 3. Tomotherapy delivers on average 20% more integral dose than IMRT and RapidArc. The volumes receiving in excess of 2 and 5 Gy are listed in Table 3. The general trend is that tomotherapy has the highest volume receiving in excess of 2 and 5 Gy, followed by RapidArc, with IMRT plans having the lowest.

### E. Treatment plan time and estimated treatment delivery time

A summary of the planning time and estimated treatment delivery time is shown in Table 4. The planning time is on average 7.5 minutes for IMRT, 48 minutes for RapidArc, and 59 minutes for tomotherapy. The estimated delivery time is on average 4.8 minutes for IMRT, 2.2 minutes for RapidArc, and 3.5 minutes for tomotherapy. The average delta value per IMRT field was determined to be 19.1±2.2 seconds per beam based on 215 head and neck IMRT fields.

**Table 4:** Treatment planning times and estimated treatment delivery times, in minutes, for plans generated for Phantoms 1–4 for IMRT, RapidArc, and tomotherapy.

		*Phantom 1*		*Phantom 2*		*Phantom 3*		*Phantom 4*	
		*Plan*	*Deliver*	*Plan*	*Deliver*	*Plan*	*Deliver*	*Plan*	*Deliver*
IMRT	5 field	6.8	3.8	6.7	3.9	6.7	4.0	7.3	4.3
	9 field	8.1	5.2	7.5	5.3	7.8	5.8	9.1	5.9
RapidArc	1 arc	45.0	1.4	38.0	1.4	30.0	1.7	53.0	1.7
	2 arc	60.0	2.8	47.0	2.8	50.0	2.8	62.0	2.8
Tomo		45.0	4.3	44.0	2.8	44.0	2.8	101.0	3.9

## IV. DISCUSSION

For all phantoms, tomotherapy was able to meet the most optimization criteria (2/4 for P1, 3/6 for P2, 2/4 for P4), followed by RapidArc (1/4 for P1, 1/6 for P2 and 0/4 for P3), followed by IMRT (0/4 for P1, 0/6 for P2 and 0/4 for P4). These results are generalized for treatment techniques and are mostly independent of the number of beams/arcs used. Tomotherapy plans were able to produce the most homogeneous dose to the PTV as an average for all phantoms $\left({\text{DHI}}_{\text{Tomo}}>{\text{DHI}}_{\text{RA}2}>{\text{DHI}}_{\text{RA}1}>{\text{DHI}}_{\text{IMRT}9}>{\text{DHI}}_{\text{IMRT}5}\right)$. This improvement in ability to meet DVH criteria comes with some costs including: longer planning time $\left(t_{\text{plan},\text{Tomo}}>t_{\text{plan},\text{RA}2}>t_{\text{plan},\text{RA}1}>t_{\text{plan},\text{IMRT}9}>t_{\text{plan},\text{IMRT}5}\right)$, longer estimated treatment times $\left(t_{\text{treat},\text{IMRT}9}>t_{\text{plan},\text{RA}2}>t_{\text{treat},\text{Tomo}}>t_{\text{treat},\text{RA}2}>t_{\text{treat},\text{RA}1}\right)$, lower conformity index $\left({\text{RCI}}_{\text{Tomo}}<{\text{RCI}}_{\text{IMRT}5}<{\text{RCI}}_{\text{IMRT}9}<{\text{RCI}}_{\text{RA}1}<{\text{RCI}}_{\text{RA}2}\right)$, and higher integral dose (approximately 1.2 times values attained for IMRT or RapidArc).

For Phantom 3, none of the treatment plans was able to meet any of the dose volume criteria. However, tomotherapy was able to provide the most dose homogeneity for that plan $\left({\text{DHI}}_{\text{Tomo}}>{\text{DHI}}_{\text{RA}2}>{\text{DHI}}_{\text{RA}1}>{\text{DHI}}_{\text{IMRT}5}>{\text{DHI}}_{\text{IMRT}9}\right)$, but less conformity $\left({\text{RCI}}_{\text{RA}2}>{\text{RCI}}_{\text{RA}1}>{\text{RCI}}_{\text{Tomo}}>{\text{RCI}}_{\text{IMRT}9}>{\text{DHI}}_{\text{IMRT}5}\right)$. There was no trend regarding the mean dose to the OARS for Phantom 3.

**Figure 6:**
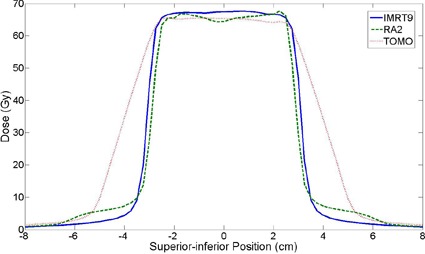
A dose profile through the geometric center of Phantom 4 with dose plotted as a function of position along the superior‐inferior direction for 9‐field IMRT (IMRT9) shown with a solid line, dual arc RapidArc (RA2) shown with a dashed line, and tomotherapy (TOMO) shown with a dotted line. Note that the PTV is within −3.75cm to +3.75cm.

This study demonstrates that treatment techniques differ in terms of the trade‐offs between treatment planning time, treatment delivery time, and overall plan quality. It is clear that sliding window IMRT treatment plans can be created in a much shorter period of time as compared to either RapidArc or tomotherapy, and that RapidArc (either single or dual beam) has the lowest estimated treatment delivery time compared to both IMRT and tomotherapy. With respect to plan quality, it appears as though tomotherapy can meet the most dose‐volume criteria and can, on average, produce plans with the most homogeneity within the target volume. The average conformity indices show that tomotherapy is worse than both IMRT and RapidArc, even though tomotherapy is thought to have much more flexibility in shaping dose distribution during optimization.^(22)^ Our result can be explained by at least two reasons. First, the two treatment planning systems (TomoTherapy and Eclipse) may have different criteria for considering whether voxels are either inside or outside of specific contours. Second, in order to deliver full dose within the target with tomotherapy, the “ramp‐up” region needs to be at least as thick as the fan beam thickness, leading to additional dose superior and inferior to the target volume.^(31)^ Superior‐inferior dose profiles through the geometric center of Phantom 4 are shown in Fig. 6 for 9‐field IMRT, 2‐arc RapidArc, and tomotherapy to illustrate the ramp‐up effect.

In this study, a single pass approach to inverse planning was undertaken as opposed to an approach where the optimization parameters are changed over multiple iterations in order to produce the plan that meets the most DVH criteria. A more robust methodology would be required to definitively rank the plan quality for IMRT, RA and Tomo. For example, by iteratively adjusting the optimization parameters the expert planner can obtain better plans, as compared to a single pass approach. In addition, it would be useful to recruit expert planners for each treatment modality. It remains to be seen what the rank order of treatment modalities would be from such a study because IMRT plans can be created in a very short period of time and many more optimization parameter changes can be exercised for IMRT as compared to RA or Tomo for a fixed planning time.

An additional limitation of a treatment planning study is that the intercomparison of data from different treatment planning systems may not be fair due to a number of complicating issues. One issue is termed the “weight paradox” by Deasy, whereby the optimal choices for the relative weights of different PTV and OAR optimization criteria are not known and may take many iterations of trial and error to determine.^(32)^ Choosing DVH criteria that are too constraining on the OAR compromises the PTV coverage, or vice versa, and may end up producing a plan that the user does not desire.^(32)^ These dosimetric obstacles have been addressed by two different approaches including multi‐criteria optimization^(^ ^33^ ^–^ ^35^ ^)^ and Pareto optimization,^(^ ^36^ ^,^ ^37^ ^)^ and when implemented in commercial treatment planning systems may be able to provide improved results. Another challenging issue is that treatment planning systems from individual manufacturers may define the objective function and weighting values for the PTV and OAR differently. This information is generally not disclosed to the user and is difficult to interpret.

One limitation in this study is that the phantoms were essentially 2D axial phantoms extended in the third dimension and do not represent contours that would be seen in a clinical environment. It is uncertain whether the TomoTherapy system would have significant difficulty in planning on phantoms with PTV and OAR contours that change shapes in the superior‐inferior direction. As mentioned in the paper by Bortfeld and Webb, within tomotherapy there is an efficiency trade‐off when planning in the superior‐inferior direction, this trade‐off involves the choices of fan beam width, modulation factor, and pitch.^(23)^ An additional study is necessary to understand how these trade‐offs impact plan quality and treatment efficiency for structure sets that vary in shape in the superior‐inferior direction.

Finally, it is difficult to know that the optimization algorithm has converged to a local minimum or how close the solution is to the global minimum. Although there are visual displays which indicate to the user how many iterations have elapsed, there isn't a criteria to stop the optimization if the optimization has converged to a set criteria such as 1% difference in objective function value change over 100 iterations. If such stopping criteria were available, then statements could be made regarding the convergence of the respective optimization algorithms.

Additional work is necessary to further understand the relative merits of differing delivery technologies for clinical geometries.

## V. CONCLUSIONS

Advanced radiation therapy delivery techniques each have their own relative merits. For the four phantoms investigated in this study, 5‐ and 9‐field IMRT treatments can be planned in the shortest time and can be delivered with the lowest integral dose. Single and dual arc RapidArc plans can be delivered in the shortest period of time and can provide the most conformal deliveries to the PTV. Finally, tomotherapy is capable of meeting most of the planning objectives and can provide the most uniform dose to the PTV.

## ACKNOWLEDGEMENTS

We would like to thank the London Regional Cancer Program (Drs. Jeff Chen and Slav Yartsev) for allowing us to use the TomoTherapy planning station for this study.
